# You read my mind: fMRI markers of threatening appraisals in people with persistent psychotic experiences

**DOI:** 10.1038/s41537-021-00173-0

**Published:** 2021-10-11

**Authors:** Raphael Underwood, Liam Mason, Owen O’Daly, Jeffrey Dalton, Andrew Simmons, Gareth J. Barker, Emmanuelle Peters, Veena Kumari

**Affiliations:** 1 King’s College London, Institute of Psychiatry, Psychology & Neuroscience, Department of Psychology, London, UK; 2 South London and Maudsley NHS Foundation Trust, Bethlem Royal Hospital, Kent, UK; 3 University College London, Max Planck Centre for Computational Psychiatry and Ageing Research, London, UK; 4 University College London, Research Department of Clinical, Educational and Health Psychology, London, UK; 5 King’s College London, Institute of Psychiatry, Psychology & Neuroscience, Department of Neuroimaging, London, UK; 6 Brunel University London, College of Health, Medicine and Life Sciences, Centre for Cognitive Neuroscience, Uxbridge, UK

**Keywords:** Human behaviour, Psychosis, Schizophrenia

## Abstract

Anomalous perceptual experiences are relatively common in the general population. Evidence indicates that the key to distinguishing individuals with persistent psychotic experiences (PEs) with a need for care from those without is how they appraise their anomalous experiences. Here, we aimed to characterise the neural circuits underlying threatening and non-threatening appraisals in people with and without a need for care for PEs, respectively. A total of 48 participants, consisting of patients with psychosis spectrum disorder (clinical group, *n* = 16), non-need-for-care participants with PEs (non-clinical group, *n* = 16), and no-PE healthy control participants (*n* = 16), underwent functional magnetic resonance imaging while completing the Telepath task, designed to induce an anomalous perceptual experience. Appraisals of the anomalous perceptual experiences were examined, as well as functional brain responses during this window, for significant group differences. We also examined whether activation co-varied with the subjective threat appraisals reported in-task by participants. The clinical group reported elevated subjective threat appraisals compared to both the non-clinical and no-PE control groups, with no differences between the two non-clinical groups. This pattern of results was accompanied by reduced activation in the superior and inferior frontal gyri in the clinical group as compared to the non-clinical and control groups. Precuneus activation scaled with threat appraisals reported in-task. Resilience in the context of persistent anomalous experiences may be explained by intact functioning of fronto-parietal regions, and may correspond to the ability to contextualise and flexibly evaluate psychotic experiences.

## Introduction

Cognitive models of psychosis propose that appraisals are key to the transition from benign psychotic experiences to clinically relevant symptoms^1–5^. These models postulate that biological, psychological, and environmental factors give rise to the aberrant assignment of salience to perceptual experiences (perceiving such experiences as more important or personally relevant than they are). By this model, cognitive appraisals of these experiences as threatening are important in generating and maintaining symptoms of psychosis. Indeed, individuals with a ‘need for care’ are more likely to appraise their psychotic experiences as caused by other people^6^, and malevolent in intent^7,8^. These threatening appraisals contribute to clinically relevant distress^9^.

There also exist healthy individuals who report persistent psychotic experiences (PEs) with little or no distress^6,10^. The Unusual Experiences Enquiry (UNIQUE) study showed that this population report enduring hallucinations in all sensory modalities, albeit with less frequency than in clinical populations with psychosis^11^. These ‘non-need-for-care’ individuals do not report the paranoia, negative symptoms, and cognitive difficulties typically found in patients^12^. Instead, these individuals appraise their experiences as significantly more ‘spiritual’ and ‘neutral’ than patients and have greater perceived control over them^9^. Alongside sociodemographic differences between those with and without a need for care, these findings support the existence of continuity between health and psychosis. These group differences have been demonstrated using well-validated questionnaires such as the Appraisals of Anomalous Experiences interview (AANEX^13^). Differences have also been shown using experimental tasks that mimic psychotic experiences such as thought interference and auditory hallucinations^11,14–16^. An example of this is the Telepath task, which mimics thought interference by giving participants the impression that a smartphone app operated by the experimenter has correctly predicted a number they have focused on in their minds. Comparing those with and without a need for care, people with a need for care report higher levels of threatening appraisals and associated distress, such as appraising the experience as being caused by another person with malign intentions^11,15^. Meanwhile, despite the presence of clinically relevant psychotic symptoms, non-need-for-care individuals are comparable to controls, appraising experiences in these tasks as neutral or even positive. The benefit of experimental tasks is that they help control for individual variation in the phenomenology of psychotic experiences and disentangle appraisal from experience. Despite this added degree of experimental control, appraisal responses, as measured by Likert scales, are subjective.

Neuroimaging techniques such as functional Magnetic Resonance Imaging^17^ (fMRI) provide an objective source of evidence that complements self-report and behavioural responses, and allow examination of neurobiological aberrations associated with threatening appraisals of anomalous experiences that might explain the transition from benign PEs to clinical symptoms and a need for care. Any neural differences observed between those with and without a need for care may highlight important protective factors in non-need-for-care individuals. Furthermore, it is as yet unknown whether non-need-for-care individuals display neural responses to anomalous experiences comparable to controls, despite similarly neutral or positive appraisals.

Threatening appraisals of anomalous experiences have not previously been examined using neuroimaging techniques but the frontal and subcortical regions commonly implicated in aberrant perception or interpretation of affect-inducing stimuli^18–21^ may be potentially relevant to appraisals. Specifically, our previous review^21^ highlighted disrupted activity and connectivity in individuals with psychosis while viewing threatening and neutral faces. These brain regions include the amygdala, insula, hippocampus, anterior cingulate cortex (ACC), parahippocampal gyrus, and prefrontal cortex (PFC). As these regions are typically involved in threat processing., these disruptions correspond to biased attention towards threatening stimuli, and an interpretative bias where neutral or ambiguous stimuli are perceived as threatening, in line with cognitive models of psychosis^2,22^.

The principal aims of this study were two-fold: One, to examine whether differences in neural (fMRI) responses to an anomalous experience-inducing task exist between individuals with persistent PEs with and without a need for care. Two, to explore whether subjective ratings of threat appraisal predict functional activation during task-induced anomalous experiences. Following on from our previous work^16^, this study used an experimental analogue of anomalous experiences to address these aims.

It was predicted that the patient (clinical) group would have significantly higher threatening appraisal scores in response to experimentally-induced anomalous experiences than the other two groups. It was further predicted that the non-need-for-care with PE (non-clinical) group would have threatening appraisal scores indistinguishable from healthy controls, in line with previous behavioural findings^11,15,16^. It was also predicted that the clinical group would show reduced activation, relative to the other two groups, in frontal and limbic regions, such as the amygdala and the prefrontal cortex, that are important for processing and appraisal of threatening or potentially threatening experiences^21^. Given that the non-clinical group report PEs but no associated distress, it was unclear what to expect at the neural level when compared to controls. Tentatively, we predicted them to show activation similar to controls, or show higher activity in some areas, such as the prefrontal cortex, that may support their neutral or a less threatening appraisal of analogous experiences. Finally, it was predicted that neural activation would scale with subjectively-rated threat appraisals.

## Results

### Sample characteristics

Demographic characteristics of the three study groups and group differences are presented in Table 1. Non-clinical participants were, on average, 13 years older than controls but not clinical participants, and with no other differences in sociodemographic variables, with the exception of employment (see Table 1). The clinical group had higher scores of depression, anxiety, and stress, and had lower IQ than did controls, but did not differ from the non-clinical group (apart from depression, which was higher in patients; see Table 1). Non-clinical participants reported significantly more years since the onset of psychotic experiences than the clinical group.

**Table 1:** Sample characteristics and statistical differences between the groups.

		Characteristics	Clinical group (*n* = 16)	Non-clinical group (*n* = 16)	Control no PE group (*n* = 16)	Significance tests
Gender (male/female)			9/7	4/12	7/9	χ^2^(2) = 3.26, *p* = 0.196
Mean age (SD)			38.87 (13.14)	45.81 (11.73)	32.71 (7.73)	F_(2,45)_ = 5.49, *p* = 0.003 (η_p_^2 ^= 0.23)^a**^
Ethnicity		White British	9	10	12	χ^2^(2) = 0.53, *p* = 0.766
		Minority ethnic groups	7	6	4	
Employment		Employed/In education	1	6	14	χ^2^(2) = 15.33, *p* < 0.001
		Not employed	15	10	2	Controls: employed > unemployed; Clinical PE: unemployed > employed
Mean years in education (standard deviation)			13.00 (2.14)	16.44 (3.90)	19.29 (5.99)	F_(2, 43)_ = 8.37, *p* = .001 (η_p_^2 ^= 0.28)^b**^
Highest level of education		University education	1	6	14	χ^2^(2) = 21.84, *p* < 0.001
		No university education	15	10	2	Controls: university > no university; Clinical PE: no university > university
Parental occupation		Professional/intermediate	9	9	13	χ^2^(2) = 2.92, *p* = 0.23 (*V* = 0.25)
		Other	7	7	3	
WASI mean (SD)		Estimated Full Scale IQ	97.87 (13.07)	112.26 (10.31)	119.50 (12.48)	F_(2, 44)_ = 13.29, *p* < 0.001 (η_p_^2 ^= 0.38)^b***^
Religious affiliation		Traditional	7	2	4	χ^2^(4) = 5.20, *p* = 0.268
		Other/spiritual	1	1	0	
		None	8	13	12	
DASS-21 mean (SD)^1^		Depression	8.87 (7.70)	2.31 (2.57)	1.73 (2.05)	F_(2, 43)_ = 10.33, *p* < 0.001 (η_p_^2 ^= 0.33)^c**, d**^
		Anxiety	6.27 (5.40)	2.88 (3.24)	2.47 (3.60)	F_(2, 43)_ = 3.78, *p* = 0.031 (η_p_^2 ^= 0.15)^c**^
		Stress	8.80 (5.92)	5.00 (3.86)	4.67 (3.42)	F_(2, 43)_ = 3.91, *p* = 0.028 (η_p_^2 ^= 0.15)^c**^
Years since onset of psychotic experiences mean (SD)^3^			15.79 (11.54)	32.53 (16.36)	-	*t*(27) = 3.16, *p* = 0.004 (*d* = 1.22)
Diagnosis (ICD-10)			Schizophrenia = 5 (31.3%) Schizoaffective = 2 (12.5%) Psychosis NOS = 3 (18.8%) F30-39 = 6 (37.5%)	-	-	-
Antipsychotic medication & dosages^2^			Medicated = 14 (87.5%) None = 2 (12.5%) Typical = 1 (6.3%) Atypical = 11 (68.8%) Clozapine = 2 (12.5%) More than 1 = 1 (6.3%)	-	-	-
Number of admissions mean [median] (range)			2.63 [1] (0–15)	-	-	-

^1^Values missing for one clinical participant and one control participant ^2^One value missing for clinical ^3^Two values missing for clinical, and one for non-clinical

Tukey’s LSD least significant difference test.

^a^Non-clinical vs. controls; ^b^Controls vs. clinical; ^c^Clinical vs. controls; ^d^Clinical vs. non-clinical.

*p < 0.05; **p < 0.01; ***p < 0.001.

Both clinical and non-clinical groups exhibited comparable levels of hallucinations and delusions on the SAPS (see Supplementary Table 1); both groups showed higher levels than the controls. With regards to negative symptoms, clinical participants reported significantly more avolition and anhedonia, and affective flattening at trend level significance, compared to the non-clinical group. In the AANEX, non-clinical participants reported significantly greater current meaning/reference, paranormal-hallucinatory experiences, and overall had more total current PEs (at trend level significance), than the clinical group (Supplementary Table 1).

### Telepath task

There was a significant main effect of Group (F(2) = 10.424, *p* < 0.001) for threatening appraisal scores (Supplementary Table 1) and this effect remained significant when we added age and IQ as covariates (F(2) = 4.300, *p* = 0.020; Age *p* = 0.09, IQ *p* = 0.86). As expected, the clinical group had higher scores for threatening appraisals than the non-clinical group (Tukey HSD *p* = 0.031) and controls (Tukey HSD *p* < 0.001), while non-clinical participants did not differ from controls (Tukey HSD *p* = 0.145). There were no group differences with regards to how globally threatening, distressing, or striking the Telepath task was (Supplementary Table 1). Similarly, there were no group differences in non-threatening appraisal scores and no participants from any group correctly guessed the task manipulation (Supplementary Table 1).

### Neuroimaging findings

**Fig. 1:**
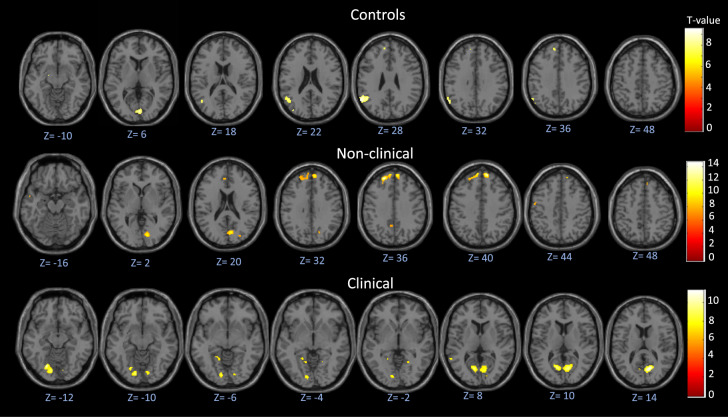
Activation patterns in each of the three study groups during the Telepath task. Contrast: Think about how this was done > Please rest. Top: significant activation in controls (N = 16). Middle: significant activation in the non-clinical group (N = 16). Bottom: significant activation in the clinical group (N = 16).

Significant task-related activations (whole-brain FWE-corrected *p* < 0.05) were observed in each of the three groups (Fig. 1, Supplementary Table 2). Whilst occipital and temporal areas showed activation in all three groups, significant activation of frontal areas was observed only in the non-clinical and control groups. Notably, the strongest activity, especially in the medial prefrontal areas, was seen in the non-clinical group (Fig. 1, Supplementary Table 2). There was no task-related deactivation observed in any group.

**Fig. 2:**
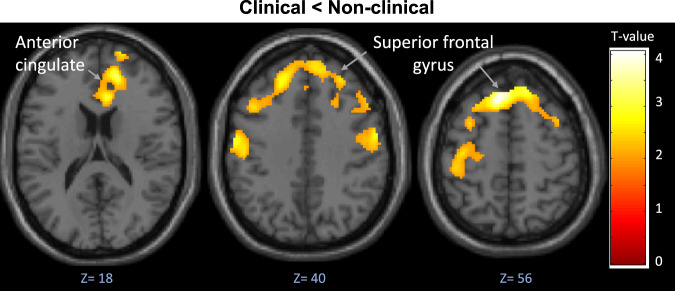
Areas showing reduced activity in the clinical group compared with the non-clinical group during the Telepath task. Cluster-forming threshold FWE-corrected *p* = 0.02; whole brain voxel threshold *p* < 0.05 uncorrected. Contrast: Think how this was done > Rest.

When the three groups were compared directly with one another, the under-recruitment of the frontal areas (bilaterally) in the clinical group, compared to the non-clinical group was confirmed (Fig. 2; whole-brain uncorrected-*p* < 0.03; Table 2). The under-recruitment of the superior-middle frontal areas (bilaterally) extending to the (right) insula was also evident in the clinical group compared to the control group but this did not survive correction).

**Table 2:** Group differences in fMRI activity (Contrast: Think how this was done > Rest; whole-brain threshold *p* < 0.05 uncorrected).

Cluster size (Voxels *n*)	Brain region	Brodmann area (BA)	Side	MNI coordinates (x y z)			Voxel *T* value		Cluster *P* value (FWE-corrected)
Clinical < Non-clinical									
6135	Superior frontal gyrus	6	L	−8	28	56		4.04	0.027
	Anterior cingulate	32	R	16	46	18		3.26	
	Superior frontal gyrus	10	R	16	64	10		3.14	
	Precentral gyrus	6	R	58	−2	36		3.15	
	Precentral gyrus	6	L	−58	−10	40		3.02	
Controls < Clinical (No sig. corrected or uncorrected clusters)									
Clinical < Controls (No sig. corrected or uncorrected clusters)									
Non-clinical < Clinical (No sig. corrected or uncorrected clusters)									
Non-clinical < Controls (No sig. corrected or uncorrected clusters)									
Controls < Non-clinical (No sig. corrected or uncorrected clusters)									

Note: Italics represent uncorrected cluster p values.

### Regression of subjective threat appraisal ratings in the PE groups

**Fig. 3:**
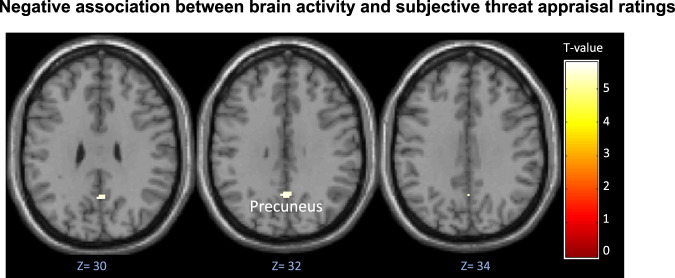
Map of precuneus activation negatively predicted by subjective threat appraisal ratings in participants with psychotic experiences. The need-for-care and non-need-for-care participants are combined. Cluster-forming threshold FWE-corrected *p* = 0.015; whole brain voxel threshold *p* < 0.05 uncorrected.

When observing both PE groups together, higher threat appraisal scores were a significant predictor of less activity in the precuneus (Fig. 3; peak: 2[x], −58[y], 32[z], *T* = 5.78; 20 contiguous voxels; cluster-forming threshold FWE-corrected *p* = 0.015). Higher threat appraisal scores did not significantly predict more activity in any areas.

## Discussion

Behaviourally, the prediction that patients would endorse significantly more threatening appraisals than the non-need-for-care (non-clinical group) and control groups was supported, as was the prediction that the latter two groups would report equivalent scores. Of note, non-clinical participants reported having had their psychotic experiences for significantly more years than the clinical group, echoing previous findings^23^.

At the neural level, the non-clinical group exhibited greater task-related activation than the clinical group (i.e. patients) across both hemispheres, primarily in frontal cortical regions, including the ACC, the inferior, middle, and superior frontal gyri. When contrasted, the non-clinical and control groups displayed equivalent activation in response to both tasks, as predicted. Finally, regressing threat appraisal scores onto task activation across groups negatively predicted cerebellar activation.

The group differences demonstrating reduced activation in patients relative to the non-clinical group were found primarily in the frontal regions that are relevant to emotional salience and regulation^24^, decision-making^25^, response selection^26^, and visual processing^27^. Prefrontal cortical regions are recruited by higher-order cognitive functions^28^, including working memory, executive control, and problem-solving. Aberrant prefrontal and ACC activity are consistently associated with a diagnosis of schizophrenia, seen as reflecting decreased affective and cognitive processing, whether observed as an increase or decrease in activation, relative to controls^29,30^. Furthermore, during tasks requiring the modulation of both attention and emotion, patients with a diagnosis of schizophrenia show impairment in fronto-limbic regions^31^. Under-recruitment of the PFC in particular has been found in schizophrenia patients when making decisions under conditions of uncertainty^32^. With these findings in mind, the frontal activation deficits observed in our clinical group may relate both to the aberrant ‘threatening’ salience postulated in cognitive models of psychosis^22,24,33^, and impaired decision making under conditions of uncertainty^34^. The deficits in higher-order cognitive function coupled with an emotionally arousing anomalous experience may have engaged reasoning biases that drive threatening appraisals. It is also worth noting that our non-clinical group showed the strongest (of all three groups; Fig. 2) medial prefrontal activity, which has previously been associated with the degree of perceived control over an unpleasant situation and long-term resilience (for a review, see^35^). It is possible that intact, or even enhanced, functioning of the frontal area enabling appropriate executive control over assessment of potentially threatening information is the main reason why non-clinical participants do not display clinically relevant symptoms despite having persistent PEs.

An alternative explanation is that the reduced activation observed in the clinical group is related to the cognitive demands of the task. The clinical group may have found the Telepath task too complex and given up quickly. All the patients in the clinical group were on antipsychotic medication, which can impact cognitive functioning^36^, while no participants in the non-clinical group had ever taken antipsychotics. The control group had a significantly higher current IQ than the clinical group (as measured by the WASI), with the non-clinical group scoring in between controls and patients. More generally, non-clinical participants have been found to exhibit higher scores on cognitive measures, relative to clinical participants^12^. Alternatively, there is evidence that this clinical population are characterised by a jumping to conclusions bias^37^, which correlates with deficits in working memory^34^, another frontal lobe-based function^38^. The jumping to conclusions bias is considered a data-gathering bias in which a decision is hastily reached without considering all the information to hand. Perhaps the clinical group quickly settled on a threatening appraisal of the task, without exercising executive control^39^, whether they found it complex or not.

Altogether, the findings in this study reinforce the validity of employing anomalous experience-inducing tasks as a method of eliciting differences in appraisals^15^. This also further supports the Garety et al. cognitive model of psychosis, which posits that maladaptive appraisals predict clinical status^2^. This result also demonstrates good validity for the Telepath task as administered in its adapted form in the fMRI environment. There have been calls for the proper integration of neurobiological data with experimental and self-report data^1^. This study represents a step in that direction.

Higher threat appraisal was associated with lower activity in the precuneus across participants with PEs. Increased activation and connectivity involving the precuneus has previously been associated with both the default-mode network and performance in a variety of cognitive tasks in healthy people^40^. In psychosis populations, reduced precuneus activation has been shown to accompany poor insight into illness and symptoms^39,41,42^ and predict poor response to Cognitive Behavioural Therapy for psychosis^43^ (CBTp), explained by the precuneus’ role in processing self-relevant information^44^. While threatening appraisals may in some way also relate to atypical self-processing, our finding of (reduced) precuneus activity and (higher) threat appraisal association might be best explained by its role in evaluating trustworthiness^45^ since threatening explanations and appraisal styles involved a degree of mistrust about others/external factors (see Supplementary Table 3 for Threatening and non-threatening explanations and appraisal styles).

Altogether, the reduced precuneus activation associated with increased threat appraisal scores, combined with the observed under-recruitment of frontal areas in our clinical group, may therefore reflect a combination of mistrust, poor self-processing, and disrupted executive control. Intact processing in these domains may therefore be relevant to understanding resilience in those reporting PEs.

First, as the study used a novel anomalous experience-inducing task within the fMRI environment, only general conclusions can be drawn regarding exactly which functions were recruited. The task, however, as expected generated a threatening appraisal and, in the control group, the area of activations also extended to include the amygdala (Fig. 2), a key emotional area^37^.

Second, the number of trials for the Telepath task was low. This was to reduce the risk of participants becoming aware of the task manipulation. Low trial numbers have previously been implemented successfully for events with high arousals, such as auditory hallucinations^46,47^. Verbal feedback during piloting and the study indicated that the task generated high attention and arousal. Additionally, the control condition closely matches the experimental condition, with the intention of controlling for non-specific activation. Third, the clinical group in this study included individuals with other psychosis spectrum disorders, since the focus was placed on symptoms over disorders during recruitment.

This study represents a successful approach to integrating neuroimaging and experimental methods of testing appraisals of anomalous experiences and is among the first to compare appraisals of anomalous experiences both at the behavioural and neural levels between individuals with persistent PEs with and without a need for care. The data revealed more threatening appraisals but lower activation in the superior and inferior frontal gyri in the clinical group, compared to the non-clinical group. Additionally, there was an association between higher precuneus activity, and lower threat appraisals across the PE groups. Taken together, these findings indicate that resilience in the context of potentially threatening anomalous experiences in people with PEs but no need-for-care may be explained by appropriate executive control, evaluation of trustworthiness (in others), and self-processing, enabled by intact functioning of frontal areas, and the precuneus. Overall, this work provides a viable design and task for analysing neural responses to experimentally-induced anomalous experiences and reinforces the cognitive model of psychosis that appraisals of anomalous experiences are key to distinguishing between individuals with psychotic symptoms with and without a need for clinical care.

## Methods

### Participants and design

The study involved three groups (see Table 1): patients with psychosis spectrum disorder (clinical), non-need-for-care participants with PEs (non-clinical), and no-PE healthy control participants, all of whom were studied on one occasion.

Sixteen patients (mean age: 39 years) with a psychosis spectrum disorder (ICD-10 diagnoses F20-39) were studied, featuring at least one psychotic episode lasting four months and currently experiencing hallucinations (at least weekly) and/or delusions (with high conviction), corresponding to a score of 3 or more on items from the Scale for the Assessment of Positive Symptoms^48^ (SAPS). Patients were recruited from outpatient services and the Psychological Interventions Clinic for Outpatients with Psychosis (PICuP) research register in the South London and Maudsley Foundation NHS Trust.

Sixteen individuals (mean age: 46 years) displaying persistent PEs without a need-for-care were studied. Individuals were included if they reported the following: at least ‘occasional’ experiences of any positive symptoms using the Unusual Experiences Screening Questionnaire [which combines the Appraisals of Anomalous Experiences Interview^13^ and the Psychosis Screening Questionnaire^49^], and a score of 3 or above on the SAPS, in the absence of self-reported drug use or altered consciousness, and whose experiences started more than 5 years previously (to minimise the likelihood of including potentially prodromal individuals). Those (*N* = 2) scoring 2 (‘unmet need’) on the ‘psychological distress’ (in relation to anomalous experiences) and ‘self-care’ dimensions of the Camberwell Assessment of Needs Short Appraisal Schedule^50^ (CANSAS) were excluded. Non-clinical participants were recruited from specialist sources using a sampling strategy developed in previous studies^15,51^, encompassing multiple sources collated into a single resource [e.g., College of Psychic Studies, The Spiritualist Association of Great Britain, New Religious Movements (NRMs), mediums, special interest websites]. Recruitment consisted of adverts disseminated via special interest group pages on the social media site Facebook, and email distribution lists provided by special interest site administrators. None of the non-clinical participants reported past or present contact with secondary care mental health services, but one participant had received treatment for depression from their General Practitioner (GP).

Sixteen controls (mean age: 33 years) scoring within 1 standard deviation of the population mean (15) or lower on the Unusual Experiences subscale of the Oxford-Liverpool Inventory of Feelings and Experiences^52^ (O-LIFE) were recruited. Online advertisements were distributed via a circular email list internal to King’s College London, visible to staff and students within the university. These advertisements were also circulated through local online forums. None had ever had contact with secondary care mental health services, but one had sought treatment for possible attention-deficit-hyperactivity-disorder from their GP.

All participants had to have a sufficient command of English, to be right-handed, as measured by the Edinburgh Handedness Inventory^53^ (scoring 50 or above), and have normal or corrected vision and hearing. It was confirmed that no participants had previous experience of the tasks. Participants were excluded if presenting with neuroimaging contraindications (i.e. claustrophobia, metal in the body, history of heart problems, pregnancy). Other exclusion criteria included a neurological history, head injury or epilepsy; primary substance dependence; pre-morbid IQ < 70, as assessed by the Wechsler Test of Adult Reading^54^ (WTAR).

The study was approved by the NHS research ethics committee (ref: 13/LO/0390). All participants provided written informed consent after the study procedures have been explained to them.

## Measures

In addition to screening measures, participants completed measures of depressive symptoms (Depression Anxiety Stress Scales^55^ (DASS-21)) and appraisals of anomalous experiences (Appraisals of Anomalous Experiences Interview^13^ (AANEX)). Current cognitive functioning was assessed using the Wechsler Abbreviated Scale of Intelligence^56^ (WASI). Previous studies have implicated IQ in the relationship between threatening appraisals of anomalous experiences^11^, and it may impact neural response to experimental tasks in patients with schizophrenia^57^. Therefore, current IQ was included as a measure of interest.

### Experimental task and procedure

The Telepath task is designed to make participants believe that their mind is being read, an analogue for thought interference. Four numbers (1–4) are presented to the participant on a smartphone screen relayed via a camera feed. Participants are asked to mentally select one number and keep it in mind. Following this, the phone is visibly placed face down, which, unbeknownst to the participant, activates an animation cycling through numbers 1–4 in 8 s intervals. Participants are asked to indicate their choice of number via a button box, while the phone is face down. Seeing this number, the experimenter then waits for the right amount of time for the participant’s chosen number to appear, then picks up the phone, revealing the participants’ chosen number. Finally, the camera feed cuts, and participants are presented with a screen displaying the words “How do you think this was done?” (see Supplementary Materials and Supplementary Figure 1 for further details on the task procedure and development).

A control condition, identical to the experimental condition, was used in which participants are assigned a number between 1 and 4, rather than being given a choice. The number participants see in the penultimate screen varies across trials between the number originally assigned to the participant, and a different number between 1 and 4. This way, participants are presented with an outcome that is not anomalous but is nonetheless unpredictable. In half of the 6 control trials, the participant’s original number is shown, with the remaining half showing a different number between 1 and 4. Overall, participants are presented with 12 trials that alternate between experimental and control conditions; 6 for the experimental condition and 6 for the control condition.

Once in the scanner, participants were familiarised with the task and button box before the task began. Participants viewed the task via a prismatic mirror fitted in the radiofrequency head coil, allowing them to see a wall projection of the experimenter’s computer screen, and pressed the appropriate button via the box provided in their right hand.

### Post-fMRI assessment of appraisals

After the functional scan, but while still laying in the scanner, spontaneous verbal explanations for the task were elicited to determine if the manipulation had been guessed correctly (e.g. “what do you think happened during the task?”). Subsequently, participants completed a Likert scale (0–10, with the following visual anchors: 0 ‘Not at all’, 2 ‘A little’, 5 ‘Somewhat’, 7 ‘Quite a lot’, and 10 ‘Extremely’), developed in a previous study^16^, asking participants to rate their conviction in seven different possible explanations (e.g. ‘It was done on purpose to trick me, or make me look stupid’). The self-report items are grouped into normalising, personalising, intentionalising, generalising, and externalising/internalising appraisal styles, following previous research^13–15^. The individual items and corresponding appraisal styles are reported in Supplementary Table 3. To derive threatening and non-threatening scores, scores on the corresponding appraisal styles were summed then averaged (threatening appraisals: external personalising/non-pesonalising, intentionalising, generalising, and non-normalising; non-threatening appraisals: external and internal normalising). A further three 10-point Likert scales with the same visual anchors were used to assess globally how striking, distressing and threatening the participants found the tasks (e.g. ‘How striking/unusual did you find the experiences?’, ‘How distressing did you find these experiences?’, ‘How threatening did you find these experiences?’). Participants utilised buttons 1 and 2 on the button box to scroll left and right respectively, pressing button 3 to confirm their choice.

### fMRI data acquisition

Echoplanar MR brain images were acquired using a 3 T GE Signa system (General Electric Healthcare, Chicago, USA). A Head-Neck-Spine (HNS) head coil was used for radiofrequency transmission and reception. In each of 40 near-axial non-contiguous planes parallel to the intercommissural (ac-pc) plane, T2*-weighted MR images depicting blood-oxygen-level-dependent contrast^17^ were acquired in an “interleaved ascending” order, with echo time (TE) = 30 ms, repetition time (TR) = 2 s, in-plane voxel size = 3.75 × 3.75 mm, slice thickness = 3.0 mm, interslice gap = 0.3 mm, and flip angle = 75°.

### Image pre-processing

For both the functional and the structural data, the origin (coordinate [0 0 0]) was set to lie on the anterior commissure, with the Y-axis parallel to the AC-PC line. The functional data were motion-corrected using a 2-pass rigid-body registration process where the images were initially aligned to the first volume, a mean image was generated and then, on the second pass, the time series was aligned to this mean image. The mean image was co-registered to a high-resolution T1-weighted structural image, and the associated affine transformations were applied to the motion-corrected time series to bring it into alignment with the T1-weighted structural image.

The parameters for warping the data to MNI stereotactic space were generated via unified segmentation of the structural image. The resultant normalization parameters were applied to the co-registered functional time series. The normalized functional data were finally smoothed with an 8 mm FWHM Gaussian smoothing kernel^58^. This is in accordance with the matched-filter theorem, to increase the signal-to-noise ratio, and render the assumptions underlying the use of gaussian random field theory and familywise error correction more valid.

### Analyses

Behavioural and questionnaire data analyses were carried out using SPSS for Windows (version 26, 2019). The α-level of significance (two-tailed) was set at *p* < 0.05 unless indicated otherwise. Appraisal scores were grouped into ‘non-threatening’ and ‘threatening’ and then averaged for analysis. The main effect of the group on threatening appraisal scores, as well as on cognitive data, was analysed using analyses of variance (ANOVAs), or the non-parametric equivalent (Kruskal–Wallis H test), depending on the results of Shapiro-Wilk normality tests. In previous studies, differences in IQ are typically not controlled for, as it may be inappropriate to co-vary for differences if these are inherent to group status^59^, as would appear to be the case with those with and without a need for care^15^). In the present study, analyses are shown with and without IQ and age added as covariates, for completeness.

fMRI data were analysed using a two-stage random effect procedure^60^. The first stage specified eight regressors encoding the onset and duration of events for trials in the experimental and control conditions. These regressors were convolved with the canonical haemodynamic response function. Following parameter estimation, a linear contrast of parameter estimates was constructed to identify brain regions during the contrast ‘Think about how this was done > Please rest’. Subsequently, task-related activations were identified (whole-brain threshold of FWE *p* < 0.05 corrected for multiple comparisons) using one-sample *t*-tests across all participants separately in the three groups. Motion parameters were also added as regressors into the model. Furthermore, the percentage of volumes whose movement in any of the motion parameters exceeded half the voxel size was calculated for each group, following Byrge & Kennedy^61^. Controls displayed 0.38% of volumes exceeding this threshold, and the clinical and non-clinical groups had comparable percentages (1.14 and 1.05% respectively). We also identified individual participants exceeding 5%, finding comparable numbers in each group (1 in the clinical group, 2 in the non-clinical group, and 1 in the control group). To determine whether these movements are likely to have influenced our key contrasts of interests, we quantified the percentage of these above-threshold movements that occurred during “Think how this was done” and “Please rest”. All bar one of these participants (in the non-clinical group) showed 0% of affected volumes for these key events. We removed this participant and repeated our analyses to compare, finding that this removal did not affect our overall pattern of results (e.g. the single precuneus cluster covariant with threat appraisal remained significant; the p-value changed from 0.018–0.024).

In the second stage, one-way ANOVA was conducted in SPM, followed by planned contrasts using *t*-tests (whole-brain threshold *p* < 0.05 uncorrected, cluster-forming threshold with family-wise error correction of *p* = 0.05). T-contrasts were created so as to observe the following potential group differences: controls > clinical, controls > non-clinical, non-clinical > clinical, non-clinical > controls, clinical > non-clinical, and clinical > controls.

This same model was then subjected to separate regression analysis, in which threat appraisal scores were added as a covariate into a second-level analysis across the two PE groups. The distribution of threat appraisal scores in controls did not overlap with those in the PE groups, hence they were excluded from this analysis. The whole-brain threshold for significance used was FWE *p* < 0.05 corrected for multiple comparisons. Identification of brain regions from MNI coordinates was completed using the software packages Talairach Client and PickAtlas^62,63^ for all results.

## Supplementary information

Supplementary Information

## Data Availability

The datasets analysed during this study are not publicly available due to participant privacy, but are available from the corresponding author on reasonable request.

## Code availability

Syntax and scripts used in SPSS and SPM respectively are not available publicly, are available from the corresponding author on reasonable request.
