# Supplementary material for: You read my mind: fMRI markers of threatening appraisals in people with persistent psychotic experiences
Source: NPJ Schizophr. 2021 Oct 11;7:49. doi: 10.1038/s41537-021-00173-0 (PMC8505497; doi:10.1038/s41537-021-00173-0)
Supplement: Supplementary file 1 — Supplementary Information [file 41537_2021_173_MOESM1_ESM.pdf]

Supplementary Materials

Design and development of the Telepath Task

The Telepath task was programmed in Visual Basic. NET and was operated via a Graphical User Interface. The experimental and control conditions are illustrated in Supplementary Figure 1.

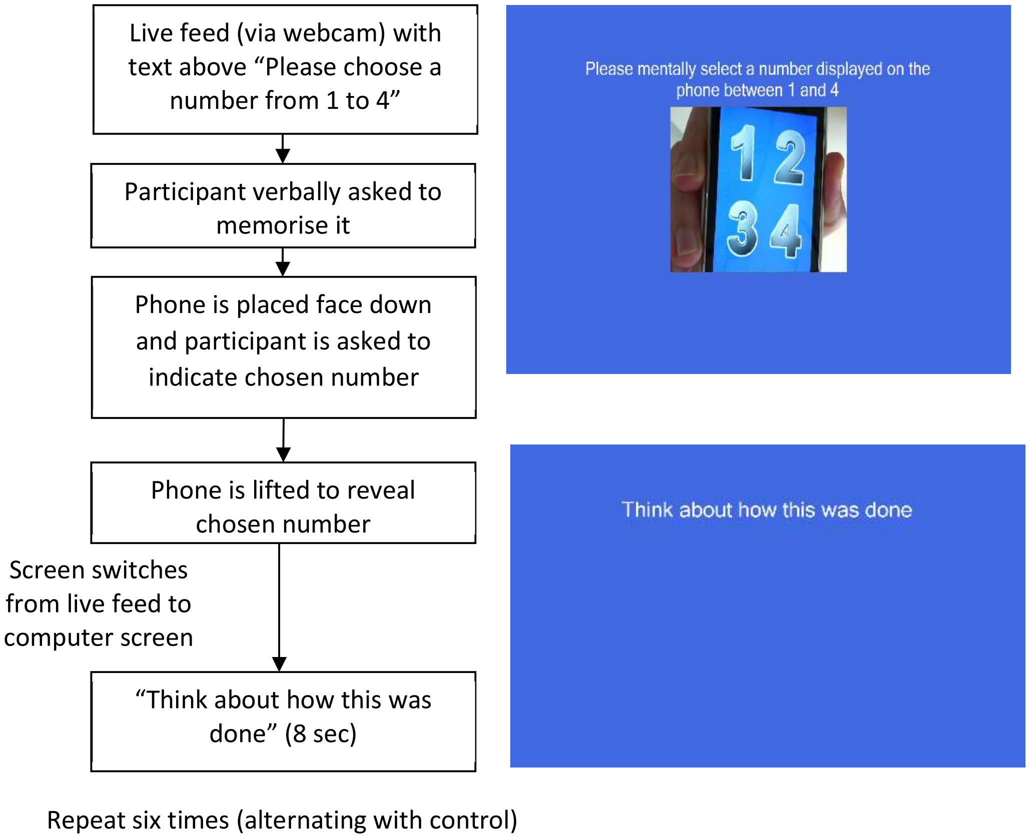

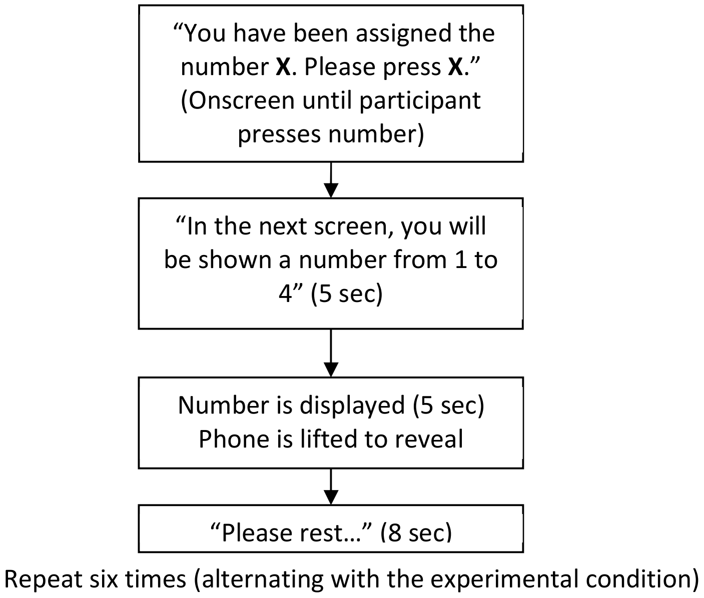

Supplementary Figure 1 Telepath task experimental and control conditions.

While the phone was face down, the experimenter waited the requisite amount of time for the desired number to appear on the phone's screen (8 sec for "1", 16 sec for "2", 24 sec for "3", and 32 sec for "4"), occupying the participant during this time by asking them to focus on their number, rehearse it, and then attempt to transmit it to the device onscreen. As a consequence of the different time lengths for each number, the experimental condition did not have a set length.

In the control condition, participants were always assigned a number between 1 and 4 at the beginning of the trial. The number subsequently displayed in the penultimate screen varied across trials between the number originally assigned to the participant, and a different number between 1 and 4. This was so that participants could not easily predict from one trial to the next which number they would be shown before the "Please rest" screen. This way, participants were presented with an outcome that was not anomalous but was nonetheless unpredictable or unexpected. Three trials showed the participant's original number, and 3 trials showed a different number between 1 and 4. Overall, participants were presented with 12 trials; 6 for the experimental condition and 6 for the control condition.

The Telepath task was initially piloted outside of the scanner to refine the above procedure. After four control participants, a number of small programming changes were made to the task regarding the wording of text appearing on screen, the stimuli presented in the control trials, and the timings of

different sections. The updated version was then re-piloted with a further five control participants. The task was also piloted with 5 non-clinical participants. Verbal feedback from the pilot study aided in refining the design and the computerised appraisal rating scales. Verbal feedback also indicated the control condition was not perceived as confusing, and did not contribute to awareness of the manipulation.

Supplementary Table 1 Summary of clinical measure scores, mean threat appraisal scores and statistical differences between groups

	Clinical (*n*=16)	Non-clinical (*n*=16)	Controls (*n*=16)	Significant group effects	Clinical vs. non-clinical	Non-clinical vs. controls	Clinical vs. controls
SAPS hallucinations	3.00 (2.00)	3.13 (0.72)	n/a	n/a	U=143, p=.590 (d=0.11)	n/a	n/a
SAPS delusions	3.25 (1.39)	3.44 (0.73)	n/a	n/a	U=125, p=.926 (d=0.02)	n/a	n/a
SAPS thought disorder	0.13 (0.50)	0.00	n/a	n/a	U=136, p=.780 (d=0.06)	n/a	n/a
SAPS bizarre behaviour	0.63 (1.20)	0.00	n/a	n/a	U=160, p=.239 (d=0.25)	n/a	n/a
SAPS inappropriate affect	0.00	0.00	n/a	n/a	N/A	n/a	n/a
SANS affective flattening	1.00 (1.41)	0.00	n/a	n/a	U=176, p=.073 (d=0.38)	n/a	n/a
SANS alogia	0.13 (0.50)	0.00	n/a	n/a	U=136, p=.780 (d=0.06)	n/a	n/a
SANS avolition	3.19 (1.83)	0.00	n/a	n/a	U=232, p<.001 (d=0.81)	n/a	n/a
SANS anhedonia	3.69 (2.21)	0.06 (0.25)	n/a	n/a	U=222, p<.001 (d=0.73)	n/a	n/a
SANS attention	2.19 (1.60)	1.81 (1.52)	n/a	n/a	U=146, p=.515 (d=0.14)	n/a	n/a
AANEX Total - Lifetime experiences	32.50 (7.14)	36.44 (5.74)	n/a	n/a	U=84, p=.102 (d=0.34)	n/a	n/a
AANEX Total - Current Experiences	28.69 (6.49)	33.00 (6.53)	n/a	n/a	U=80, p=.073 (d=0.38)	n/a	n/a
Total AANEX score	59.50 (12.07)	66.75 (11.40)	n/a	n/a	U=79, p=.067 (d=0.38)	n/a	n/a

AANEX - Meaning/reference - Current	6.13 (1.71)	9.13 (2.22)	n/a	n/a	U=37.5, p<.001 (d=0.71)[1]	n/a	n/a
AANEX - ‘Paranormal-Hallucinatory’- Current	4.50 (1.67)	6.56 (1.83)	n/a	n/a	U=54, p=.004 (d=0.58)[1]	n/a	n/a
AANEX - ‘Cognitive-Attention’- Current	4.63 (1.96)	3.69 (1.20)	n/a	n/a	U=160.5, p=.224 (d=0.25)	n/a	n/a
AANEX - ‘Dissociative-Perceptual’- Current	4.88 (1.86)	3.94 (1.34)	n/a	n/a	U=164.5, p=.171 (d=0.29)	n/a	n/a
AANEX - First Rank Symptoms -Current	9.00 (2.73)	9.81 (2.40)	n/a	n/a	U=102.5, p=.341 (d=0.20)	n/a	n/a
Threatening appraisals							
	2.84 (1.94)	1.59 (1.03)	0.68 (0.76)	F(2)=10.424, p<.001 (η^2_p=0.32)	Tukey HSD p=.031	Tukey HSD p=.145	Tukey HSD p<.001
Non-threatening appraisals							
	3.13 (1.42)	4.00 (1.23)	3.71 (1.72)	F(2)=1.45, p=.245	n/a	n/a	n/a
Global striking							
	5.00 (3.48)	4.63 (3.01)	4.19 (2.34)	F(2)=0.30, p=.744	n/a	n/a	n/a
Global threat							
	2.13 (3.07)	0.44 (0.73)	0.69 (1.14)	H(2)=5.22, p=.074	n/a	n/a	n/a
Global distress							
	2.31 (3.24)	0.44 (0.81)	1.13 (1.82)	H(2)=4.40, p=.111	n/a	n/a	n/a

[1]Non-clinical were sig. higher than clinical.

Note: All scores for SAPS & SANS items are global scores.

Legend: SAPS = Scale for the Assessment of Positive Symptoms (Andreasen 1984); SANS = Scale for the Assessment of Negative symptoms (Andreasen 1983); AANEX = Appraisals of Anomalous Experiences Interview (Brett et al. 2007).

Supplementary Table 2 Task-related activations (Think about how this was done > Please rest) in the control, non-clinical (no need for care) and clinical PE (patient) groups (*FWE* $p<0.05$). No area showed significant deactivation any group

Cluster size (Voxels *n*)	Brain region	Brodmann area (BA)	Side	MNI coordinates (x y z)			Voxel *T* value	Cluster *P* value (corrected for multiple comparisons)
Control group								
286	Superior temporal gyrus	39	L	-60	-56	28	9.52	<.001
	Supramarginal gyrus	39	L	-56	-62	32	8.63	
	Middle temporal gyrus	39	L	-42	-62	18	7.63	
82	Cuneus	17	R	4	-84	6	8.67	<.001
23	Superior frontal gyrus	8	L	-8	48	36	7.61	0.003
10	Superior occipital gyrus	19	L	-36	-82	22	7.92	0.010
1	Amygdala	N/A	L	-18	-6	-10	7.39	0.036
Non-clinical group								
429	Cuneus	18	R	10	-74	20	10.59	<.001
	Lingual gyrus	18	R	14	-80	2	9.92	
218	Superior frontal gyrus	9	L	-20	40	36	14.68	<.001
	Medial frontal gyrus	8	L	-6	52	40	10.49	
	Middle frontal gyrus	9	L	-22	38	36	7.89	
160	Superior frontal gyrus	8	R	14	50	40	14.53	<.001
10	Precentral gyrus	4	L	-52	-6	44	8.23	0.011
10	Middle temporal gyrus	21	L	-52	0	-16	8.11	0.011

Clinical group								
377	Cuneus	18	R	14	-68	14	11.33	<.001
233	Declive	N/A	L	-22	-72	-12	10.50	<.001
	Lingual gyrus	18	L	-12	-82	-10	9.14	
111	Cuneus	30	L	-12	-68	10	9.45	<.001
22	Parahippocampal gyrus	19	L	-20	-48	-4	7.75	0.004
	Culmen	N/A	L	-14	-54	-4	7.33	
11	Middle temporal gyrus	21	L	-60	-48	8	7.63	0.010
5	Parahippocampal gyrus	19	R	20	-56	-2	7.14	0.020

Supplementary Table 3 Threatening and non-threatening explanations and appraisal styles for the experimental task

Non-threatening appraisals	
External - Normalising	'It is just a simple number puzzle'
Internal - Normalising	'It is to do with natural extrasensory perception (ESP)/psychic or paranormal abilities'
Threatening appraisals	
External - Personalising	'It was not just about the phone; there is someone behind the scenes involved'
External - Non-personalising	'It works because the system is able to read people's minds.'
External - Intentionalising	'It was done on purpose to trick me, or make me look stupid'
External - Generalising	'It is a trick that is part of a bigger conspiracy'
Internal - Non-normalising	'This means that something is wrong with me'
